# Editorial: Community series in crosstalk in ferroptosis, immunity and inflammation, volume II

**DOI:** 10.3389/fimmu.2026.1942493

**Published:** 2026-08-11

**Authors:** Hao Wang, Matheus Silvério Mattos, Weijia Zhang

**Affiliations:** 1 Department of Nutrition and Food Hygiene, School of Public Health, Zhengzhou University, Zhengzhou, China; 2 Laboratory of Molecular Immunology, Department of Microbiology, Immunology and Transplantation, Rega Institute for Medical Research, KU Leuven, Leuven, Belgium; 3 Institute of Metabolism and Cell Death, Helmholtz Zentrum München, Neuherberg, Germany

**Keywords:** cancer, ferroptosis, immunity, inflammation, sepsis

Ferroptosis, a novel form of regulated cell death triggered by iron accumulation and lipid peroxidation, plays critical roles in diverse tissue injuries. In recent years, immune dysfunction caused by ferroptosis has emerged as a key driver in numerous severe pathological conditions, including sepsis and cancer (1, 2). However, the mechanisms underlying ferroptosis-mediated regulation of immune functions remain poorly understood.

The previous Research Topic, “Crosstalk in Ferroptosis, Immunity & Inflammation: Volume I,” consisted of a series of articles focusing on the mechanisms by which ferroptotic processes intersect with immune responses and inflammatory pathways (3). The current “Crosstalk in Ferroptosis, Immunity & Inflammation: Volume II” extends this scope with 23 new studies investigating the interplay between ferroptosis and immune responses, and elucidating inflammatory processes in diseases such as sepsis, neurological disorders, inflammatory bowel diseases, bone disorders, cardiovascular disease and cancer. These studies provide novel insights into potential therapeutic targets and strategies of pharmacological interventions.

Sepsis is a dysregulated host response to infection leading to life-threatening organ dysfunction, with the lung and heart being particularly vulnerable, and it is associated with high global mortality. The pathogenesis of sepsis is complex, and effective therapeutics remain limited (4). Whether ferroptosis contributes to sepsis, especially in the process of immune regulation and tissue injury, has attracted extensive attention. Using integrated bioinformatics and machine learning approaches, Luo et al. identified and validated several diagnostic biomarkers (PGD, MAPK14, and KRAS) for neonatal sepsis associated with ferroptosis and cuproptosis pathways. Wang et al. explored the relationship between ferroptosis and fibroblast growth factor-2 (FGF2) in the pathogenesis of sepsis-related acute lung injury, and revealed the therapeutic potential of targeting the FGF2-mediated inhibition of ferritinophagy-induced ferroptosis. Given the lack of biomarkers for precisely characterizing lung-specific pathological changes, Dang et al. elucidated the regulation of cell-specific exosomes in alveolar-capillary barrier and proposed exosomes as promising diagnostic biomarkers. Neutrophilic asthma is a severe phenotype of asthma with unclear mechanisms. Song and Wang proposed a hypothesis-generating framework suggesting ferroptosis-associated metabolic dysregulation may contribute to neutrophilic asthma, and highlighted ferroptosis-targeting agents and metabolic interventions as potential therapeutic strategies. Moreover, Li et al. synthesized existing evidence to propose that ferroptosis acts as a critical regulatory hub in the pathological network of periodontitis.

Ferroptosis has emerged as the key contributor to neuropathological diseases. Neurodegenerative diseases are characterized by the progressive loss of neuronal structure and function and include major disorders such as Alzheimer’s disease (AD) and Parkinson’s disease (PD) (5). Yi et al. summarized the regulatory mechanisms of ferroptosis in AD, and proposed potential therapies targeting ferroptosis via small-molecule inhibitors or natural compounds to slow or halt AD progression. Tu et al. focused on the relationship between the PD-related genes and ferroptosis, and suggested ferroptosis as a potential therapeutic target and early diagnostic marker for PD. Exosomes are nanoscale extracellular vesicles capable of crossing the blood-brain barrier. When exosomes deliver ferroptosis-regulatory cargos such as microRNAs, long non-coding RNAs, and proteins, they may acquire the ability to modulate ferroptosis in neurological injuries. Bao et al. proposed exosomes as precision delivery platforms for ferroptosis-targeted therapy in neurological disorders. Ferroptosis is not restricted to neurons but can also occur in immune cells. Abnormal activation and functional changes of immune cells induced by ferroptosis may reshape the inflammatory-immune microenvironment in brain and contribute to central nervous system (CNS) diseases. Li et al. systematically summarized the core biological features of ferroptosis and immune responses, emphasizing their crosstalk in major CNS disorders. Temporal lobe epilepsy (TLE) is a common CNS disease frequently resistant to pharmacotherapy. Huang et al. demonstrated that CPEB1 aggravates neuronal injury in TLE by driving ferroptosis–neuroinflammation crosstalk, and targeting this pathway represents a promising therapeutic strategy for drug-resistant TLE. Pain is a category of neurological disorders. Yan et al. summarized the involvement of ferroptosis in pain responses, and proposed that alleviating lipid peroxidation and iron accumulation to relieve pain may serve as potential therapeutic strategies for pain-related disorders.

Accumulating evidence indicates that ferroptosis interacts with innate immune signaling pathways in macrophages, contributing to chronic inflammation and inflammatory diseases, including inflammatory bowel disease (IBD) and arthritis (6, 7). Chen et al. summarized the distinct mechanisms of ferroptosis in macrophages, and discussed the functional roles of macrophage ferroptosis in the development of IBD and inflammation-associated tumorigenesis. Li et al. identified the natural compound 5-O-Methylvisammioside (MeV) as a promising candidate for colitis treatment. In animal models of colitis, MeV treatment maintained epithelial barrier integrity by activating Nrf2/HO-1 to suppress ferroptosis. In bone homeostasis, Xiao et al. outlined the mechanisms and therapeutic prospects of ferroptosis in the bone microenvironment and common bone/joint diseases. Knee osteoarthritis (KOA) is a chronic inflammatory joint disorder. Using single-cell transcriptomics integration, Wu et al. identified novel mechanisms by which ferroptosis modulates immune microenvironment remodeling and immunometabolic dysfunction in KOA. Additionally, Wang et al. summarized the roles of ferroptosis in the development and treatment of rheumatoid arthritis.

Cardiovascular disease (CVD) is the leading cause of mortality and disability worldwide. Ferroptosis has emerged as a common factor in multiple CVDs, such as atherosclerosis, drug-induced heart failure, myocardial ischemia-reperfusion injury, sepsis-induced cardiomyopathy and diabetic cardiomyopathy (8). Notably, the ferroptotic cell types vary across these CVDs, including cardiomyocytes, endothelial cells and immune cells. Wang et al. summarized the intricate network linking ferroptosis and immune-mediated inflammation in CVDs, emphasizing the mechanisms by which ferroptosis modulates immune cell function, inflammatory cytokine release, and oxidative stress. Macrophage polarization modulates arterial plaque formation, and protecting M2 macrophage against ferroptosis may alleviate atherosclerosis (9). Through bioinformatics analysis and *in vitro* experiments, Wu et al. identified several key regulatory genes which drive macrophage ferroptosis to reshape the immune microenvironment and modulate atherosclerosis. Miao et al. revealed that Vitamin D supplementation inhibits ferroptosis in cardiomyocytes and ameliorated diabetic cardiac injury. Beyond CVD, Luo et al. linked ferroptosis to various major kidney diseases, highlighting the critical role of ferroptosis in the initiation and progression of renal disorders.

The roles of ferroptosis in tumor biology are multifaceted. In tumor cells, pharmacological induction of ferroptosis has emerged as a potential anticancer strategy. In immune cells, ferroptosis may impair the anti-tumor immunotherapy efficacy, resulting in immune suppression, antigen presentation defects, and remodeling of the tumor immune microenvironment (10). Li et al. summarized the current understanding of ferroptosis-related mechanisms underlying immune evasion in cervical cancer, including alterations in ferroptosis regulators, redox imbalance, and ferroptosis-induced release of immunomodulatory molecules. Xu et al. discussed the dual function of circRNAs in ferroptosis and anti-tumor immunity, and proposed circRNA as potential diagnostic biomarkers and novel therapeutic targets in cancer. Therefore, further investigation is required to determine how to selectively induce ferroptosis in tumor cells while sparing immune cells.

Through experimental investigations and literature reviews, this Research Topic advances our understanding of the crosstalk between ferroptosis regulatory pathways and immune functions. Future research should further elucidate the regulation of ferroptosis in distinct immune cell subsets at different developmental stages, and facilitate the development of precise strategies to modulate ferroptosis in specific immune cell populations. The elucidation of ferroptosis mechanisms in immune cells will also provide critical insights into novel therapeutic targets and strategies for diseases such as sepsis and cancer.
